# Supplementary material for: HIVprotI: an integrated web based platform for prediction and design of HIV proteins inhibitors
Source: J Cheminform. 2018 Mar 9;10:12. doi: 10.1186/s13321-018-0266-y (PMC5845081; doi:10.1186/s13321-018-0266-y)
Supplement: Supplementary file 2 — Additional file 2. Source code of HIVProtI web server. [file 13321_2018_266_MOESM2_ESM.zip › HIVprotI_Source-code/hivprotI/weka-3-6-6/doc/weka/classifiers/rules/RuleStats.html]

RuleStats


---


|  |  |  |
| --- | --- | --- |
| **Overview** \| **Package** \| **Class** \| **Tree** \| **Deprecated** \| **Index** \| **Help** \| **Weka's home** | |  |
| **PREV CLASS**   **NEXT CLASS** | **FRAMES**    **NO FRAMES**     **All Classes** |  |
| SUMMARY: NESTED \| FIELD \| CONSTR \| METHOD | DETAIL: FIELD \| CONSTR \| METHOD |  |


---


## weka.classifiers.rules Class RuleStats

```
java.lang.Object
  weka.classifiers.rules.RuleStats
```

**All Implemented Interfaces:**: java.io.Serializable, RevisionHandler

---

``` public class RuleStats extends java.lang.Object implements java.io.Serializable, RevisionHandler ```

This class implements the statistics functions used in the
propositional rule learner, from the simpler ones like count of
true/false positive/negatives, filter data based on the ruleset, etc.
to the more sophisticated ones such as MDL calculation and rule
variants generation for each rule in the ruleset.

Obviously the statistics functions listed above need the specific
data and the specific ruleset, which are given in order to instantiate
an object of this class.

**Version:**
:   $Revision: 4608 $

**Author:**
:   Xin Xu (xx5@cs.waikato.ac.nz)

**See Also:**: Serialized Form

---

**Constructor Summary**

`RuleStats()`
  
          Default constructor

`RuleStats(Instances data,
FastVector rules)`
  
          Constructor that provides ruleset and data


| **Method Summary** | |
| --- | --- |
| `void` | `addAndUpdate(Rule lastRule)`             Add a rule to the ruleset and update the stats |
| `void` | `cleanUp()`             Frees up memory after classifier has been built. |
| `double` | `combinedDL(double expFPRate, double predicted)`             Compute the combined DL of the ruleset in this class, i.e. |
| `void` | `countData()`             Filter the data according to the ruleset and compute the basic stats: coverage/uncoverage, true/false positive/negatives of each rule |
| `void` | `countData(int index, Instances uncovered, double[][] prevRuleStats)`             Count data from the position index in the ruleset assuming that given data are not covered by the rules in position 0...(index-1), and the statistics of these rules are provided.  This procedure is typically useful when a temporary object of RuleStats is constructed in order to efficiently calculate the relative DL of rule in position index, thus all other stuff is not needed. |
| `static double` | `dataDL(double expFPOverErr, double cover, double uncover, double fp, double fn)`             The description length of data given the parameters of the data based on the ruleset. |
| `Instances` | `getData()`             Get the data of the stats |
| `double[]` | `getDistributions(int index)`             Get the class distribution predicted by the rule in given position |
| `Instances[]` | `getFiltered(int index)`             Get the data after filtering the given rule |
| `java.lang.String` | `getRevision()`             Returns the revision string. |
| `FastVector` | `getRuleset()`             Get the ruleset of the stats |
| `int` | `getRulesetSize()`             Get the size of the ruleset in the stats |
| `double[]` | `getSimpleStats(int index)`             Get the simple stats of one rule, including 6 parameters: 0: coverage; 1:uncoverage; 2: true positive; 3: true negatives; 4: false positives; 5: false negatives |
| `double` | `minDataDLIfDeleted(int index, double expFPRate, boolean checkErr)`             Compute the minimal data description length of the ruleset if the rule in the given position is deleted.  The min\_data\_DL\_if\_deleted = data\_DL\_if\_deleted - potential |
| `double` | `minDataDLIfExists(int index, double expFPRate, boolean checkErr)`             Compute the minimal data description length of the ruleset if the rule in the given position is NOT deleted.  The min\_data\_DL\_if\_n\_deleted = data\_DL\_if\_n\_deleted - potential |
| `static double` | `numAllConditions(Instances data)`             Compute the number of all possible conditions that could appear in a rule of a given data. |
| `static Instances[]` | `partition(Instances data, int numFolds)`             Patition the data into 2, first of which has (numFolds-1)/numFolds of the data and the second has 1/numFolds of the data |
| `double` | `potential(int index, double expFPOverErr, double[] rulesetStat, double[] ruleStat, boolean checkErr)`             Calculate the potential to decrease DL of the ruleset, i.e. |
| `void` | `reduceDL(double expFPRate, boolean checkErr)`             Try to reduce the DL of the ruleset by testing removing the rules one by one in reverse order and update all the stats |
| `double` | `relativeDL(int index, double expFPRate, boolean checkErr)`             The description length (DL) of the ruleset relative to if the rule in the given position is deleted, which is obtained by:   MDL if the rule exists - MDL if the rule does not exist   Note the minimal possible DL of the ruleset is calculated(i.e. |
| `void` | `removeLast()`             Remove the last rule in the ruleset as well as it's stats. |
| `static Instances` | `rmCoveredBySuccessives(Instances data, FastVector rules, int index)`             Static utility function to count the data covered by the rules after the given index in the given rules, and then remove them. |
| `void` | `setData(Instances data)`             Set the data of the stats, overwriting the old one if any |
| `void` | `setMDLTheoryWeight(double weight)`             Set the weight of theory in MDL calcualtion |
| `void` | `setNumAllConds(double total)`             Set the number of all conditions that could appear in a rule in this RuleStats object, if the number set is smaller than 0 (typically -1), then it calcualtes based on the data store |
| `void` | `setRuleset(FastVector rules)`             Set the ruleset of the stats, overwriting the old one if any |
| `static Instances` | `stratify(Instances data, int folds, java.util.Random rand)`             Stratify the given data into the given number of bags based on the class values. |
| `static double` | `subsetDL(double t, double k, double p)`             Subset description length:   S(t,k,p) = -k\*log2(p)-(n-k)log2(1-p) Details see Quilan: "MDL and categorical theories (Continued)",ML95 |
| `double` | `theoryDL(int index)`             The description length of the theory for a given rule. |

**Methods inherited from class java.lang.Object**

`equals, getClass, hashCode, notify, notifyAll, toString, wait, wait, wait`

**Constructor Detail**

### RuleStats

```
public RuleStats()
```

:   Default constructor

---


### RuleStats

```
public RuleStats(Instances data,
                 FastVector rules)
```

:   Constructor that provides ruleset and data

    **Parameters:**: `data` - the data: `rules` - the ruleset


**Method Detail**

### cleanUp

```
public void cleanUp()
```

:   Frees up memory after classifier has been built.

---


### setNumAllConds

```
public void setNumAllConds(double total)
```

:   Set the number of all conditions that could appear
    in a rule in this RuleStats object, if the number set
    is smaller than 0 (typically -1), then it calcualtes
    based on the data store

    :   **Parameters:**: `total` - the set number

---


### setData

```
public void setData(Instances data)
```

:   Set the data of the stats, overwriting the old one if any

    :   **Parameters:**: `data` - the data to be set

---


### getData

```
public Instances getData()
```

:   Get the data of the stats

    :   **Returns:**: the data

---


### setRuleset

```
public void setRuleset(FastVector rules)
```

:   Set the ruleset of the stats, overwriting the old one if any

    :   **Parameters:**: `rules` - the set of rules to be set

---


### getRuleset

```
public FastVector getRuleset()
```

:   Get the ruleset of the stats

    :   **Returns:**: the set of rules

---


### getRulesetSize

```
public int getRulesetSize()
```

:   Get the size of the ruleset in the stats

    :   **Returns:**: the size of ruleset

---


### getSimpleStats

```
public double[] getSimpleStats(int index)
```

:   Get the simple stats of one rule, including 6 parameters:
    0: coverage; 1:uncoverage; 2: true positive; 3: true negatives;
    4: false positives; 5: false negatives

    :   **Parameters:**: `index` - the index of the rule **Returns:**: the stats

---


### getFiltered

```
public Instances[] getFiltered(int index)
```

:   Get the data after filtering the given rule

    :   **Parameters:**: `index` - the index of the rule **Returns:**: the data covered and uncovered by the rule

---


### getDistributions

```
public double[] getDistributions(int index)
```

:   Get the class distribution predicted by the rule in
    given position

    :   **Parameters:**: `index` - the position index of the rule **Returns:**: the class distributions

---


### setMDLTheoryWeight

```
public void setMDLTheoryWeight(double weight)
```

:   Set the weight of theory in MDL calcualtion

    :   **Parameters:**: `weight` - the weight to be set

---


### numAllConditions

```
public static double numAllConditions(Instances data)
```

:   Compute the number of all possible conditions that could
    appear in a rule of a given data. For nominal attributes,
    it's the number of values that could appear; for numeric
    attributes, it's the number of values \* 2, i.e. <= and >=
    are counted as different possible conditions.

    :   **Parameters:**: `data` - the given data **Returns:**: number of all conditions of the data

---


### countData

```
public void countData()
```

:   Filter the data according to the ruleset and compute the basic
    stats: coverage/uncoverage, true/false positive/negatives of
    each rule

---


### countData

```
public void countData(int index,
                      Instances uncovered,
                      double[][] prevRuleStats)
```

:   Count data from the position index in the ruleset
    assuming that given data are not covered by the rules
    in position 0...(index-1), and the statistics of these
    rules are provided.  
    This procedure is typically useful when a temporary
    object of RuleStats is constructed in order to efficiently
    calculate the relative DL of rule in position index,
    thus all other stuff is not needed.

    :   **Parameters:**: `index` - the given position: `uncovered` - the data not covered by rules before index: `prevRuleStats` - the provided stats of previous rules

---


### addAndUpdate

```
public void addAndUpdate(Rule lastRule)
```

:   Add a rule to the ruleset and update the stats

    :   **Parameters:**: `lastRule` - the rule to be added

---


### subsetDL

```
public static double subsetDL(double t,
                              double k,
                              double p)
```

:   Subset description length:   
    S(t,k,p) = -k\*log2(p)-(n-k)log2(1-p)
    Details see Quilan: "MDL and categorical theories (Continued)",ML95

    :   **Parameters:**: `t` - the number of elements in a known set: `k` - the number of elements in a subset: `p` - the expected proportion of subset known by recipient **Returns:**: the subset description length

---


### theoryDL

```
public double theoryDL(int index)
```

:   The description length of the theory for a given rule. Computed as:  
    0.5\* [||k||+ S(t, k, k/t)]  
    where k is the number of antecedents of the rule; t is the total
    possible antecedents that could appear in a rule; ||K|| is the
    universal prior for k , log2\*(k) and S(t,k,p) = -k\*log2(p)-(n-k)log2(1-p)
    is the subset encoding length.

    Details see Quilan: "MDL and categorical theories (Continued)",ML95

    :   **Parameters:**: `index` - the index of the given rule (assuming correct) **Returns:**: the theory DL, weighted if weight != 1.0

---


### dataDL

```
public static double dataDL(double expFPOverErr,
                            double cover,
                            double uncover,
                            double fp,
                            double fn)
```

:   The description length of data given the parameters of the data
    based on the ruleset.

    Details see Quinlan: "MDL and categorical theories (Continued)",ML95

    :   **Parameters:**: `expFPOverErr` - expected FP/(FP+FN): `cover` - coverage: `uncover` - uncoverage: `fp` - False Positive: `fn` - False Negative **Returns:**: the description length

---


### potential

```
public double potential(int index,
                        double expFPOverErr,
                        double[] rulesetStat,
                        double[] ruleStat,
                        boolean checkErr)
```

:   Calculate the potential to decrease DL of the ruleset,
    i.e. the possible DL that could be decreased by deleting
    the rule whose index and simple statstics are given.
    If there's no potentials (i.e. smOrEq 0 && error rate < 0.5),
    it returns NaN.

    The way this procedure does is copied from original RIPPER
    implementation and is quite bizzare because it
    does not update the following rules' stats recursively
    any more when testing each rule, which means it assumes
    after deletion no data covered by the following rules (or
    regards the deleted rule as the last rule). Reasonable
    assumption?

    :   **Parameters:**: `index` - the index of the rule in m\_Ruleset to be deleted: `expFPOverErr` - expected FP/(FP+FN): `rulesetStat` - the simple statistics of the ruleset, updated if the rule should be deleted: `ruleStat` - the simple statistics of the rule to be deleted: `checkErr` - whether check if error rate >= 0.5 **Returns:**: the potential DL that could be decreased

---


### minDataDLIfDeleted

```
public double minDataDLIfDeleted(int index,
                                 double expFPRate,
                                 boolean checkErr)
```

:   Compute the minimal data description length of the ruleset
    if the rule in the given position is deleted.  
    The min\_data\_DL\_if\_deleted = data\_DL\_if\_deleted - potential

    :   **Parameters:**: `index` - the index of the rule in question: `expFPRate` - expected FP/(FP+FN), used in dataDL calculation: `checkErr` - whether check if error rate >= 0.5 **Returns:**: the minDataDL

---


### minDataDLIfExists

```
public double minDataDLIfExists(int index,
                                double expFPRate,
                                boolean checkErr)
```

:   Compute the minimal data description length of the ruleset
    if the rule in the given position is NOT deleted.  
    The min\_data\_DL\_if\_n\_deleted = data\_DL\_if\_n\_deleted - potential

    :   **Parameters:**: `index` - the index of the rule in question: `expFPRate` - expected FP/(FP+FN), used in dataDL calculation: `checkErr` - whether check if error rate >= 0.5 **Returns:**: the minDataDL

---


### relativeDL

```
public double relativeDL(int index,
                         double expFPRate,
                         boolean checkErr)
```

:   The description length (DL) of the ruleset relative to if the
    rule in the given position is deleted, which is obtained by:   
    MDL if the rule exists - MDL if the rule does not exist   
    Note the minimal possible DL of the ruleset is calculated(i.e. some
    other rules may also be deleted) instead of the DL of the current
    ruleset.

    :   **Parameters:**: `index` - the given position of the rule in question (assuming correct): `expFPRate` - expected FP/(FP+FN), used in dataDL calculation: `checkErr` - whether check if error rate >= 0.5 **Returns:**: the relative DL

---


### reduceDL

```
public void reduceDL(double expFPRate,
                     boolean checkErr)
```

:   Try to reduce the DL of the ruleset by testing removing the rules
    one by one in reverse order and update all the stats

    :   **Parameters:**: `expFPRate` - expected FP/(FP+FN), used in dataDL calculation: `checkErr` - whether check if error rate >= 0.5

---


### removeLast

```
public void removeLast()
```

:   Remove the last rule in the ruleset as well as it's stats.
    It might be useful when the last rule was added for testing
    purpose and then the test failed

---


### rmCoveredBySuccessives

```
public static Instances rmCoveredBySuccessives(Instances data,
                                               FastVector rules,
                                               int index)
```

:   Static utility function to count the data covered by the
    rules after the given index in the given rules, and then
    remove them. It returns the data not covered by the
    successive rules.

    :   **Parameters:**: `data` - the data to be processed: `rules` - the ruleset: `index` - the given index **Returns:**: the data after processing

---


### stratify

```
public static final Instances stratify(Instances data,
                                       int folds,
                                       java.util.Random rand)
```

:   Stratify the given data into the given number of bags based on the class
    values. It differs from the `Instances.stratify(int fold)`
    that before stratification it sorts the instances according to the
    class order in the header file. It assumes no missing values in the class.

    :   **Parameters:**: `data` - the given data: `folds` - the given number of folds: `rand` - the random object used to randomize the instances **Returns:**: the stratified instances

---


### combinedDL

```
public double combinedDL(double expFPRate,
                         double predicted)
```

:   Compute the combined DL of the ruleset in this class, i.e. theory
    DL and data DL. Note this procedure computes the combined DL
    according to the current status of the ruleset in this class

    :   **Parameters:**: `expFPRate` - expected FP/(FP+FN), used in dataDL calculation: `predicted` - the default classification if ruleset covers null **Returns:**: the combined class

---


### partition

```
public static final Instances[] partition(Instances data,
                                          int numFolds)
```

:   Patition the data into 2, first of which has (numFolds-1)/numFolds of
    the data and the second has 1/numFolds of the data

    :   **Parameters:**: `data` - the given data: `numFolds` - the given number of folds **Returns:**: the patitioned instances

---


### getRevision

```
public java.lang.String getRevision()
```

:   Returns the revision string.

    :   **Specified by:**: `getRevision` in interface `RevisionHandler`
    :   **Returns:**: the revision


---


|  |  |  |
| --- | --- | --- |
| **Overview** \| **Package** \| **Class** \| **Tree** \| **Deprecated** \| **Index** \| **Help** \| **Weka's home** | |  |
| **PREV CLASS**   **NEXT CLASS** | **FRAMES**    **NO FRAMES**     **All Classes** |  |
| SUMMARY: NESTED \| FIELD \| CONSTR \| METHOD | DETAIL: FIELD \| CONSTR \| METHOD |  |


---
